# Extra Supplement—The Nursing Mirror

**Published:** 1891-02-28

**Authors:** 


"The Hospital" Nursing Mirror.

Being the Extra Nursing Supplement of "The Hospital" Newspaper.

Contributions for this Supplement should be addressed to the Editor, The Hospital, 140, Strand, London, W.C., and should have the word "Nursing" plainly written in left-hand top corner of the envelope.

### En Passant.

BEDFORDSHIRE INSTITUTE.—Mr. Whitbread, M.P., took the chair at the annual meeting of Governors of the Bedford Institute of Hospital Trained Nurses. The report was satisfactory and an improvement on the last; the Institute seems now to have fairly mastered the financial difficulty, and to be well on the road to success. All sections of the Bedford inhabitants benefit, for there is a district nurse to attend the very poor, and the lowest fee for a private nurse by the week is one guinea.

THE SISTERS OF ST. JOHN.—The report for 1890 of the work done by the nursing community of St. John the Divine, is, as usual, heartstirring reading. The hospital at Lewisham has treated 264 patients; the private nurses from 68 and 70, Drayton Gardens, have nursed 661 paying patients, as well as a large number of the poor in West Chelsea, Poplar, Lewisham, and Deptford. At the Lying-in Hospital, in Gunter Grove, the patients numbered 102 during the year, and the mid-wife has attended 149 out-door cases. St. Gabriel's Hospital for Infants has been amalgamated with the Victoria Hospital—a very wise move. No wonder with all this work on hand that the sisters appeal for funds. The community is under the patronage of Earl Nelson and Sir Edmund Hay Currie.

HAMPSHIRE NURSES' INSTITUTE.—The annual meeting of this Institute was held on February 10th, under the presidency of Surgeon-General Maclean. The Chairman submitted the annual report, which showed that the earnings of the nurses for the year amounted to £610 13s., an excess over the previous year of £26 2s. 8d. The house-keeping expenses was less by £10 18s. 9½d. The debt on the Home has been quite cleared off. The report spoke very highly of the work carried on by the district nurses, who had been in 19 parishes, making 7,222 visits to the sick poor, the Matron making 40 such visits, and the private nurses 12. It was hoped that the parishes would lend the Institute some small amount of pecuniary assistance to meet the cost of this work. The Committee thanked Mrs. Edwin Jones for the means of hiring a piano, for the use of the nurses when resting from their labours, for three months. The private nurses number 14 and three probationers in training. Surgeon-General Maclean said he believed there ought always to be 30 nurses in a home to make it pay. Dr. Trend spoke in favour of the nurses and their work.

A NEW SOCIETY.—A meeting was held at Denbigh on February 9th, with the object of starting a district nurse. One or two ladies had taken trouble to ascertain that the Queen's Jubilee Institute were prepared to hand over for the use of Denbigh the following sums, viz., £40 for the first year, £20 for the second, and £10 for the third, towards the maintenance of a district nurse, in order to secure which it was necessary that an affiliated branch of the institute should be established. The expense of such a nurse would amount to about £80 per annum. Subscriptions to the amount of £25 were immediately promised. Miss Gee explained that at present the services of a thoroughly trained Welsh-speaking nurse were available, and as this was not always the case it was desirable that the opportunity be taken advantage of. On the motion of Mr. T. J. Williams, seconded by Mr. J. Parry Jones, the following were appointed the committee of management: The Mayor, the Rector, the seven medical men in practice in Denbigh, and one representative from each of the following bodies: Methodists, Wesleyans, Independents, Baptists, and Roman Catholics.

SHORT ITEMS.—The Local Government Board have appointed Dr. Bridges and Mr. R. Hedley to hold an official inquiry on oath into the complaints against the management of the Eastern (Homerton) Hospital of the Metropolitan Asylums Board.—The annual report of the Coppice Lunatic Asylum concludes thus: "There have been few changes among the attendants and servants, and their conduct generally has been satisfactory; and the manner in which they, for the most part, have performed their irksome and onerous duties has been most praiseworthy."—Dr. Grace Davenport, one of the five women physicians of Texas, was recently appointed first assistant of the Insane Asylum at Terrell, in that State.—A correspondent of the *British Medical Journal* says the best artificial sponge is made of Hartmann's wood-wool enclosed in a layer of sublimate gauze.—The members of the Wesleyan Chapel at Newcastle maintain a district nurse, who, during last year, attended 155 patients; there is also a deaconess engaged in rescue work.—About 150 of the Manchester nurses were entertained lately at tea, followed by charades, by Mrs. and Miss Marsden.—The Bishop of Ely and Lady Alwyn Compton were present at the annual meeting of the St. John District Nurses at Worcester. The report was most satisfactory.

A WOMAN'S LIFE.—The following is a short account of the nursing career of one of the first thousand of R.N.P.F. for Nurses. "When but a child I was never so happy as when I could sit by or be with a sick person, but the great ambition of my life was to nurse the sick and wounded soldiers. I was left an orphan when scarcely fourteen years of age, but my way did not become clear to enter a hospital till the November of 1880, when I was taken as a yearly pupil nurse at Crumpsall Infirmary, Manchester. I cannot express to you my joy when I found myself within its walls, and the first evening I was called nurse, and the first evening I was allowed to attend a lecture, how I drank in all I heard. Of course I was to be on a month's trial to see if I was in earnest, but it was no passing fancy with me. I was determined if I did not suit it would not be my fault. I longed for that month to pass. I must have been impatient, and when it did end I did not wait for my matron to tell me what she thought of me as a pro., but I went to her office and said, "please do you think I will suit?" I think I see her dear face now as she looked up at me and smiled, and asked me if I really liked the work. I told her I loved it, so it was all right, and I stayed one year at Crumpsall, and I never spent a happier year in my life. I was taught medical, surgical, midwifery, and fever work, passing through children's and adults' wards. From Manchester, I went to the S. A. Home for Nurses, Oxford, nursing either rich or poor as I was called upon to do. I remained there close on three years, but as I wanted to see a little more of the world I joined the late Holland Institute for English Nurses, in Paris, and when I had been there about six months I was sent on to the Branch Home at Nice. I nursed for this good Institute for two years, and was very fortunate in being sent about. I not only had cases in various parts of France, but nursed in Switzerland, and Italy; and, apart from the Holland Institute, I nursed one season at beautiful Cannes. And now the ambition of my childhood is about to come to pass. I return to England. I apply (I pray) for the post of Nursing Sister in Her Majesty's service. I am accepted. I go to the R. V. Hospital, Netley. When there sixteen months, I get my orders for foreign service, and proud was I when I found myself on the steamer to bring me to Egypt, and since being here I have had the honour of being sent on active service, and have really nursed the sick and wounded from the battle-field, and achieved my life's ambition."

### Lectures on Surgical Ward Work and Nursing.

By Alexander Miles, M.B. (Edin.), C.M., F.R.C.S.E.

### Lecture XV.—DRESSING TRAYS AND ANÆSTHETICS.

Instrument Trays.—I hope to say something later on the subject of instruments, and the setting of them out for operation.

Hot and Cold Water, etc.—Under the table should be kept a large tin pail furnished with a close-fitting cover, and filled with hot water, with which to dilute and heat up the various lotions. A supply of cold water will also be useful. In addition a large, wide-mouthed slop-pail should be ready into which dirty lotions, etc., may be emptied. Under the operating table a large, shallow tray for dirty dressings should be placed, or a wide box containing sawdust.

Syringes.—The uses and methods of employing the various forms of syringe have already been treated of (page lxxii), and I need not repeat what I have there said.

(2) Dressings Table.—On this table are two distinct sets of articles, which are under the care of different assistants; one the dressings, the other the requirements of the anæsthetist.

The *contents of the Operation Dressing Tray* differ very slightly, if at all, from those of the ward dressing tray already described. They are: 1, a supply of plain lint; 2, boracic lint; 3, gutta-percha tissue; 4, oiled silk protective; 5, carbolised gauze bandages; 6, double cyanide bandages; 7, domette bandages; 8, cotton bandages; 9, adhesive plaster; 10, four clove-hitch garters; 11, safety pins; 12, wool boxes, containing (*a*) corrosive wool, (*b*) wood wool.

*Anæsthetic Tray.*—The preparation and arrangement of this will be left to the nurse, although of necessity, the contents are mainly used by a doctor, or medical student superintended by a qualified man. Contents: 1, two bottles chloroform; 2, two bottles ether; 3, one bottle "A.C.E." mixture; 4, chloroform drop-bottle; 5, inhaler or folded towel; 6, small pot of vaseline for face; 7, tongue forceps; 8, clean towel; 9, solution tin; 10, hypodermic syringe charged with ether; 11, several hypodermic syringes (uncharged); 12, bottle of Eucalyptus oil for sterilising these; 13, two minim glasses; 14, supply of brandy; 15, strong solution of ammonia; 16, solutions of cocaine, 5 per cent., 10 per cent., 20 per cent.

The Anæsthetic.—Although it is seldom required of a nurse that she should be able to administer anæsthetics, it is only right that she should have a general idea of the action of these, the dangerous conditions arising during their administration, and, above all, the means to be adopted of treating these conditions. As every one knows, chloroform and ether, the most commonly employed anæsthetics are given mainly to prevent the patient suffering pain, whether it be the pain of a surgical operation or of parturition. They are also given sometimes to overcome muscular action; for example, in the reduction of dislocations in very strong men. No patient should have either of these substances administered to him, unless he has been properly "prepared" for it, except, of course, in cases of accident where this is obviously impossible. I shall have more to say on this preparation of patients for anæsthetics subsequently. I shall first speak of *Chloroform*, and direct your attention to three important points with which you should be acquainted regarding it—1, its physiological action; 2, the proper method of administration; 3, the dangers likely to arise, and their treatment.

(1) Physiological Action.—Chloroform first acts by stimulating the nervous system, and then by depressing it; there is an early period during which the patient has delusions, and struggles violently, and then follows a period of stupor, with relaxation of muscles and blunting of the sensations. You must bear in mind, however, that chloroform poisons the brain centres in a definite order, and that you only desire this process to go a certain length, within which the patient is safe. The order is as follows: 1, Paralysis of voluntary motion; 2, paralysis of sensation; 3, paralysis of reflexes (to be attained); 4, paralysis of respiration; 5, paralysis of heart (to be avoided). Sometimes accidents occur which apparently disarrange this regularity of action, but these are due to some intercurrent condition, and not to chloroform itself. Having attained the first three stages, you must remember that only a very little more is needed to reach the remaining two, and that your patient is in a very dangerous condition, and one which requires your undivided attention. Chloroform, being a cumulative poison, should be administered with extreme caution, as the patient may have taken a good deal more than is indicated by his general condition. Fortunately, however, it is also a volatile poison, so that, should you have overstepped the limit of safety, by carrying on artificial respiration for a sufficient time to enable the excess to volatilize, the respiratory and cardiac centres may resume their functions.

(2) Method of Administration.—Let me say, to begin with, that the chloroformist has quite enough to do with his own work, and must on no account try to assist with anything else, or to see the operation. The patient when thoroughly "under" is at the brink of the grave, and a single step may place him beyond recall. Before beginning the administration, the patient should be made quite comfortable, lying on his back with a hard pillow under his head. Make certain that he has nothing in his mouth before beginning. I have seen one patient choke on a piece of tobacco, and another on a set of false teeth. Such things, then, should be removed. Everything about his neck and waist must be quite loose to facilitate respiration. Bear in mind that chloroform is a heavy vapour, and so falls on to the patient's face, irritating the skin. To prevent this irritation, smear a thin layer of vaseline, or some such ointment, over the face. No special inhaler is necessary, a towel folded into a square and placed over the face being quite sufficient. Some surgeons, however, prefer a apparatus of some sort or another. Allis' ether inhaler is very suitable for chloroform, as it admits a sufficient quantity of air to mix with the vapour, and is also saving of the anæsthetic. A simple inhaler is made of a small wire mask, over which is stretched a layer of thick flannel. Success in administering chloroform, however, depends, not on the apparatus used, but on the man using it. A few drops of chloroform are sprinkled over the surface of the towel, and the patient is asked to take long breaths. Children usually hold their breath and refuse to take the chloroform, and the best way to manage is to tell them to "blow it away," or to get them to cry, when the resulting deep inspiration effects the desired end. Don't hurry the patient much at first, and should he feel inclined to choke, remove the towel for a second or two and give him a breath of fresh air. Always remember that chloroform is only safe when mixed with about equal parts of air. When the patient is struggling it is not advisable to resist him, as it only increases the trouble. Guide his movements, and the struggling stage will be quickly passed. Patients who have been addicted to alcohol usually present exaggerated struggling phenomena, with sometimes tonic or clonic rigidity of the whole body lasting for a few seconds, and sometimes even epileptiform attacks. When once the struggling stage is passed in these cases it does not recur.

How are you to know that your patient is under? No one sign is infallible, and you must test him in several ways to make sure: (*a*) *Muscular relaxation* indicates that he is at least partly under. Test this by raising a limb and seeing if it drops limp when unsupported. (*b*) *Local sensibility* may be tested by pricking or pinching the skin, say, at the seat of operation. (*c*) *Conjunctival reflex:* This is a very handy test, although by no means a reliable one. It consists in observing if the eyelids close when the conjunctiva is touched. With the pulp of the middle finger gently and completely raise the upper eyelid, then lightly touch the conjuctiva with the tip of the forefinger. As a rule, if the eyelids are not forcibly closed, the patient may be considered deeply under; but often the reflex is present when the patient is dangerously anæsthetised, and *vice versa*. It is particularly unsatisfactory as a test in children.

While the operation is going on the chloroformist must watch the respiration and the general appearance of his patient. He can best watch the respiration by holding his hand over the patient's mouth and feeling the hot air at each expiration. The heaving of the chest is also an indication, but it is sometimes simulated by contractions of the diaphragm which are unconnected with the respiratory act. The breathing can also be heard in most cases, but this test is not to be relied upon, especially when the spray is being used. The patient is in a dangerous condition when the breathing is shallow and irregular, or gasping and sighing. Stertorous breathing is an indication that enough has been given although not a sign of danger. Don't suppose that because a patient is breathing he is all right; attend to other signs. If he stops breathing he is at the point of death. After a little experience in giving chloroform the general appearance of the patient gives you a good idea of his condition. If his face has its natural colour, with red lips, he is fairly well, but if, on the other hand, he become livid or pallid, with blue lips and upturned eyes he is in a dangerous condition. You are often told to watch the pulse. It may be useful as indicating the general state of the patient as regards shock, but as an indication of the action of the chloroform it is of no use. To watch for the pulse stopping is to watch for the patient's death.

### Princess Christian's Daughter.

MISS E. DURHAM, Farringford, Freshwater, Isle of Wight, acknowledges the following additional subscriptions towards a wedding present for Princess Louise of Schleswig-Holstein to be given as a proof of the gratitude of nurses for the interest Princess Christian has ever taken in their progress. Subscriptions will be received until the end of March, and will be acknowledged in these pages. From February 16th to February 23rd the following sums were received:—

*Matrons* (5s. each): Miss Cooper, Miss Millard. *Sisters* (2s. 6d. each): Sister Annie Edwards, Sister Sully, Sister Mary, M. Sandford. *Nurses* (1s. each): Ella, Georgina Arthur, Wool, Lonston, Stuart, Rhodes, Rachel, M. E. B., Elizabeth Clark, Ada Deeth, M. A. S., Emma Willey, M. Heg, R. Maunder, M. E. Robinson, H. Hawkins, Flora E. Sundden, E. Rogerson, J. S. Woolley, H. E. Cestin, E. Wright, M. Hudson, M. Radford, M. H. Stevens, E. Allen, C. Blackwell, and E. G.

### Presentations.

ON February 7th, Miss Crofton was presented by Sir Robert Heron with an address and a handsome dressing bag. Miss Crofton has for 22 years acted as Lady Superintendent of the Bird's Nest, Kingstown, a convalescent home for the poor children of Dublin. Miss Crofton has now resigned, and the above presentation was made on behalf of the committee and friends of the institution to show their appreciation of her services.

MISS PHILIPPA HICKS has been presented with a handsome Tennyson in a case, and an illuminated address, by the Committee, General and Medical, of the Hospital for Sick Children, Great Ormond Street. The address says the book is presented as a token of personal appreciation, as well as an acknowledgment of the work done by Miss Hicks as Lady Superintendent of the Hospital.

### A Winter's Holiday.

### IV.—IN SPAIN.

OUR drive over the mountains into Spain was delightful and wonderful; it really was extraordinary how the driver of the diligence steered it through the very narrow, crooked streets of Arnegay, the frontier town. Part of the journey we rode on ponies, and from Rounceveaux to Bourgette we walked over a great silent plain. The sun was very hot and burnt our faces, but there was a delicious breeze. Presently we came in sight of the solitary inn of Bourgette. At the door stood a group of figures, two of whom speedily advanced to meet us, one being our prospective host and the other an aged Spanish traveller.

Imagine our amazement and delight when English voices also greeted us, and in the other members of the party we discovered some real friends of our own. A Scotch doctor, with his wife and sister, all well known to us in London! He was here on business connected with some mines in the neighbourhood, in which his wife had lately inherited shares of considerable value. Hence his planning of what was turning out a pleasant tour for all three of them, and a well-earned holiday for himself. The sight of his kindly face was indeed welcome to us, his friends; and to how many a sick man and woman has not that wise brain and good heart brought comfort? Aye! and renewed health where possible. A great man in his profession, a humble man in his own eyes, served "for love, not fear." "Long life to Dr. Lindsay" is the students' cry whenever a festivity gives excuse for the cheers which youth loves to utter . . . . . By the exclamations of our friends, now and afterwards, we first realized that we two English women were doing an unprecedented thing! Probably never before had ladies crossed those mountains without a proper male escort; we might think ourselves lucky to have reached Bourgette in safety, &c., &c. Privately we thought that two such quiet, unremarkable women, accustomed to act for themselves for the last ten years, were little likely to get into any position from which they could not extricate themselves. However, for Dr. Lindsay's opinion we had sufficient respect to give him a patient hearing, and when he wound up with, "My dear ladies, you are not in London!" and "You don't know the country!" we were silenced, if not convinced.

It was pleasant to sit down and enjoy a decent cup of tea brewed by Mrs. Lindsay, whilst we talked over our various experiences.

The heat of the sun, and the fatigue of the ride, had rather upset Frances, and she went to bed very soon, thus escaping dinner. This was lucky, as the universal sauce of hot *oil* was hardly calculated to suit the palate of a person already feeling seedy! The spirit lamp and kettle, without which we never travelled, enabled her to have some soup later on, thanks to one of those many "meat essences" which are so useful in emergencies.

A cup of herb tea was taken up to her by the landlady whilst I sat at dinner, and poor Frances felt too weary and bad to maintain for long her strong objection to swallow it. For "the sake of peace and quietness," as she assured me afterwards, she had gulped down the nastiest decoction she had ever tasted in her life!

Dr. Lindsay roared with laughter when this tale reached his ears, and he vowed that it was capital experience for a nurse who was more often the administrator than the victim in her own person of such like nauseous draughts.

During the evening I discovered that the diligence by which we had intended to pursue our journey on the morrow would not pass till the following morning at 3 a.m., so we had an extra day to spend at Bourgette. The presence of our English friends made the delay not unwelcome.

As they and the host, who talked French though his wife could not, and the other Spaniards at the dinner-table seemed quite regretful that we had missed seeing the Rounceveaux relics, we agreed to walk over there in the morning.

### Notes from Australia.

(*By Our Own Correspondent.*)

Melbourne, January 7th.

One of the things in Melbourne that has not before been mentioned in The Hospital is the excellent Nurses' Home now situated in Alma Road, St. Kilda. It is the outcome of an enterprise that was commenced in Hobart, Tasmania, by Miss Roynton. Not succeeding in Hobart, the "Home" was removed to a Melbourne suburb called Prahran, without any immediate good result. Four years since, Mrs. Sergeant was appointed Lady Superintendent, and was unusually fortunate in having the active support of some of the most capable and influential ladies in the community. The "Home" was removed to a pleasanter suburb called St. Kilda, and every effort was made to give good grounds for reversing the previous popular verdict of "failure." Complete success has followed. There are thirty nurses at present on the staff, many of them of English training, and the home has become self supporting. Besides board and lodging, each nurse receives from the Committee a salary of from £40 to £45, and an addition of 25 per cent. from her earnings. Indoor uniform is provided, and must be worn; out of doors the dress is optional. All nursing appointments are made by the Lady Superintendent, who can have no higher eulogy than the fact that all things work harmoniously, and that she enjoys the confidence of nurses, doctors, and patients alike. No nurse is received who does not undertake to remain on the staff for as long as a year. It was pleasant to see The Hospital lying on the office table, and to learn that sundry of the nurses were members of the Nurses' National Pension Fund.

Perhaps it is unnecessary to add that there is no difficulty in maintaining a full and efficient staff at this very well-to-do institution; but if any of our nurses contemplate emigration to Australia, we say, Take with you all possible skill and resource and elasticity, but be sure to leave at home all idea that you may possibly be in a position to patronise or instruct.

The Charities Commission has gone on tour through the provinces, and means to visit all the larger hospitals without giving previous notice. They will probably, by these means, obtain a better insight into hospital management than by sitting in a room and listening to witnesses contradicting one another.

General Booth's scheme has aroused great interest amongst the charitable here, and great enthusiasm; but our Premier is protesting against the "colony over sea" being established under his jurisdiction. All the Premiers will probably do the same thing, not seeing the absurdity of grudging these bountiful tracts of waste land to poor earth-starving creatures from the old country.

Our Christmas appeals were responded to very liberally, over £7,000 being given through the *Argus* newspaper alone. The Medical Society of Victoria held two successful meetings during December, and the British Medical Association lately met to hear an interesting paper from Dr. D. A. Gresswell.

A thrilling tale of the sea is told by the crew of the *Kelton*, of Glasgow, which lately went ashore near Point Nepean. There were 31 men on board, and 20 got small-pox; one died. It is believed that the germs were shipped together with some ballast at Rio Janeiro. There are still six men ill, and they have been landed at a quarantine station, and a doctor and two nurses are attending to them.

It is difficult to turn one's attention away from this big busy town of Melbourne, but the Children's Hospital at Adelaide certainly deserves some mention and must be interesting to your readers, for many London nurses have worked there. The hospital treated 286 in-patients and 3,916 out-patients last year, at a total cost of £3,360. The resident staff consists of a House Surgeon, Matron, Head Nurse, trained nurse, two senior nurses, and five junior nurses. The Head Nurse is Miss Noble, who last year succeeded Miss Hill. The nurses get good training, and, as a rule, take to private work as soon as their time is up. What do you sticklers of etiquette think of the fact that the medical staff give free lectures, not only to the nurses, but to any of the town dwellers who choose to attend? The Board of Management consists of an equal number of gentlemen and ladies, and there is also a large Ladies' Committee, which does good work. Women serve on committees very largely in Australia.

### Examination Questions.

Forty answers were received to the last question—"How would you apply and dress a blister?"—and not one of these was incorrect. It has, therefore, been most difficult to decide who merits the prize, but, after much consideration, we have awarded it to Nurse Rachel Parsons, of Brighton, to whom we have sent a copy of Murrell's well-known work on "Masso-Therapeutics." Answers worthy of honourable mention were sent by Nurse F. Lang, Nurse Weedon, Nurse E. Correll, Nurse Alice Watson, Nurse A. B. Baillie, Sister Gardiner, Nurse Emma Andrew, and Nurse Helen Wilson. We note in Nurse Maggie Stock's answer that she says with regard to the dressing, "Be sure and have it ready beforehand, and do not keep the patient waiting while you go off to get it." The following is a quotation from Nurse Margaret White's answer: "A small blister can be quickly raised by putting some cotton-wool into a silver thimble, and pouring on it a few drops of ammonia; invert the thimble on skin and keep it there for fifteen minutes." Nurse Maude Fletcher thus describes Dr. John Williams' method of dry dressing: "Dress with iodoform wool and strapping which should be left on until it comes away without dragging the skin." The question for next month is: "If a child is seized with croup what should be done while waiting for the doctor?" Answers must reach this office (140, Strand) by March 9th; the word "Nursing" must be written on the envelope. Only nurses are eligible for this competition, and each answer must be written on one side of the paper only (any sized paper will do), and must be accompanied by the writer's full name and address.

Prize Answer.—Blisters are employed to produce surface inflammation, in order to reduce deeper-seated inflammation and pain. Usually a blistering fluid or plaster is used (Cantharides, or Spanish fly), which has the effect of raising the cuticle of the skin and drawing into it serum from the blood. The exact size and shape of blister, and the exact spot to be acted on, should be defined by the doctor. When ordered to be applied to any joint, it is more effectual when placed in the region of the joint than immediately over it, so, for the hip joint place in the groin; for the knee or elbow joint apply in two "half-moon" shapes above and below the part; for the ankle put it between that and the heel. Fluid is preferable to plaster, as the latter will drag on the surrounding parts as the bleb rises. Shave off any hairs on the part defined, and wash well with hot soapy water to remove dirt and natural oil. Keep your hands away from your eyes when applying any blistering material. Sometimes the natural oil will prevent the bleb rising, then paint on a little ether. Sometimes a thin tissue paper is placed between the blister and the skin, if the skin is delicate. If a plaster is used it should be secured by a bandage, not strapping, as that would increase the dragging and cause unnecessary pain. When the cuticle is risen remove the plaster very gently without breaking the visicle. If fluid is ordered, mark an outline round the defined spot with olive oil or cut a hole in paper the required size, and hold firmly over the spot so that the blister may be limited; paint on with camel-hair brush. Be careful not to drop any fluid anywhere than the spot given, as quickly wiping off will not prevent an unnecessary blister. Its rising progress must be watched, and if very slow, a warm linseed or bread poultice should be applied. It will take from six to twelve hours to rise, the time depending on the age, thickness of skin, and general condition of patient. To dress the blister a small receiver or test tube should be placed under it to catch the serum, then snip it at its most dependent part, gently press over the bleb and see that all t he fluid escapes; take care it does not get on any other part, and keep it for examination. Dress with olive oil or zinc ointment spread on lint, keep clean and renew dressing twice or more in 24 hours. If ordered to keep the blister open, remove the cuticle entirely and, unless it is desirable to obtain a more powerful counter irritant (perhaps savine or mercurial ointment) which will be ordered, spread a piece of lint, the exact size of wound, with simple dressing olive oil or zinc ointment and apply, over which put a larger piece of lint and secure with a bandage.

### Everybody's Opinion.

[*Correspondence on all subjects is invited, but we cannot in any way be responsible for the opinions expressed by our correspondents. No communications can be entertained if the name and address of the correspondent is not given, or unless one side of the paper only be written on.*]

### THE DEATH RATE IN TYPHOID.

Miss Hall writes: Miss Fletcher's apposite letter on "The Death Rate in Typhoid" raises a question which must have occurred to many. A comparison of the percentage of recoveries in hospital with that of those nursed privately would not only be interesting, but, if all the conditions were given, eminently useful. Especially would this be the case if details of nursing were given, so that it might be seen whether the doctor's directions, more particularly in relation to rest and abstinence from solid food, had been rigidly adhered to. That implicit obedience is not always rendered to a medical man's orders we all know. Especially is this apt to be the case in the early stages of convalescence from typhoid, when the patient craves and begs for that food which kindly but weak-minded relatives consider it cruel to refuse, and yet which is so often the real cause cf disappointing relapses whose source baffles inquiry. That so-called trained attendants are not always alive to the duty of strict obedience is illustrated by a case which came under my own observation. A young girl was taken ill at school, of what proved to be typhoid. She was removed to the school "hospital," the mother sent for, and a trained nurse engaged at, I think, £2 2s. per week. The milk supply being suspected as a possible source of infection, orders were given that the patient should have none which had not first been boiled. This precaution, the mother discovered, was not being taken, and, on remonstrating with the nurse, she was met by the remark that to boil the milk was "only a doctor's fad." Far be it from me to under-rate the services of trained nurses—I have known too many not to fully recognise their value and self-sacrificing devotion to duty—but this question of training is one which needs thorough ventilation. Let us have training by all means, but let it be of the right sort. Nowadays it is the fashion to sneer at the phrase "a born nurse." Yet there is a large measure of truth in it, for, just as no amount of extraneous heat will vivify a marble statue, so all the training in the world cannot make a nurse (in the true sense of the term) out of one who has not the requisite qualifications, even though she may be able to perform, and have passed a creditable examination in, the technical routine of her calling. The letter of a "Fever Nurse" throws a side light upon this question, and helps us to see how it is that there are signs of a growing preference on the part of doctors and patients for an intelligent person who, if technically "untrained," will yet do as she is told.

### NURSES' HOME, TRURO.

The Hon. Secretary writes: "I am sure that you do not wish to make your paper a medium for misleading your readers, and I trust, therefore, that you will kindly correct in your next issue, some of the suggestions made with reference to the above Home in your paper of February 14th. The scale of our nurses' salaries so far from being low, is certainly above the average, that is from £25 to £35, except in the case of young nurses who have just left the hospital. The arrangements of the Home under the care of our Superintendent are most comfortable, and I need scarcely say that laundry work forms no part of the occupation of our nurses."

### A SOURCE OF DANGER.

Arthur J. Wilmshurst writes: What poor suffering humanity would do without the hospitals one hardly cares to contemplate, for it is to these noble institutions that many owe their present health. And then to have homes in the country or at the seaside where the patients can thoroughly establish their health at little or no expense to themselves is indeed a great boon. With what taste, too, are these buildings designed; the flights of stone steps that leading up to the entrances, the tesselated vestibules and polished floors, all, doubtless, make a great impression on visitors. Many large convalescent homes are under the care of sisters, who treat their patients with great kindness and forbearance; these homes are very nice places, the beds are comfortable, the meals regular, and the sanitary arrangements are good, but the floors are often very dangerous. In some homes the floors are waxed and polished so highly that it is difficult to keep from slipping down; the passages, too, are often tiled, becoming sometimes very slippery. If those whose legs are sound find the floors dangerous, to the unfortunate cripples the danger must be much greater. To one of these seaside homes there is attached a chapel, which must have cost a large sum to build and furnish. On the two sides and at the back of the altar are handsome stained glass windows, while the floor is a most exquisite piece of workmanship. The design is worked out in tiles, some of which are in strips, and shiny; besides the tiles, there are the artistic iron gratings so often seen in church floors. The church is lighted by means of embellished brass gas brackets; and leading up to the altar are two or three handsome marble steps. But with all this beauty, there is one thing wrong, and that is, the floor is slippery. Should a patient chance to place his crutch on those narrow tiles, it will be a miracle if he did not slip down, while to avoid the slippery places is not very easy. If some means were adopted that would make the floors safer, especially for the cripples, these homes would be most excellent places; but a slip or a tumble means a great shock to the patient, if nothing worse followed.

### Notes and Queries.

To Correspondents.—1. Questions may be written on post-cards. 2. Advertisements in disguise are inadmissible. 3. In answering a query please quote the number. 4. If a private answer is desired, a stamped addressed envelope must be forwarded.

Notice.—We must really ask our correspondents to be more particular in enclosing name and address. Constantly we receive queries or letters with no name attached.

### Queries.

(36) *Carbolic.*—Is carbolic acid the best disinfectant in scarlet fever nursing?

(37) *Hydropathics.*—Are there any hydropathics which are moderate in their charges, and suitable for a gentleman recovering from rheumatic fever? How about Limpley Stoke?—*A. G. F.*

### Answers.

(34).—The simplest way is to use a pneumonia bandage made of flannel, fastens over the chest, and has straps over the shoulder which can be crossed in front.—*K. A.*

*Brown.*—Probationers of 35 years of age can only receive training by paying a guinea a-week. Apply to the London Hospital, Whitechapel, or to the Boston Hospital, Lincolnshire.

*A Subscriber.*—The Maternity Hospital, Manchester, or the Ladies' Lying-in Charity, Liverpool.

*Nurse M.*—A patient recovering from scarlet fever, and still undergoing "peeling," is not disinfected by any amount of carbolic. Until the process of peeling is finished there is risk of infection. But a nurse who has attended a patient can be thoroughly disinfected by means of carbolic. See query.

*Nurse Winter.*—The Earlswood Asylum, free by election, or by payment according to circumstances. Or the Eastern Counties Asylum, Station Road, Colchester; by election, or by payment of £50 a-year. Write to the Secretary of the last.

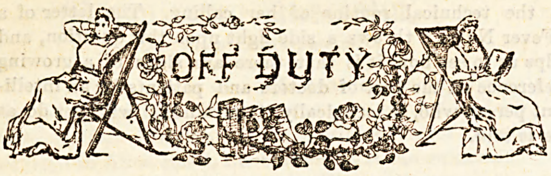

### Nurse Hilary.

(*Continued from page* cxviii.)

Day after day passed, and the outward life of the Eleanor Ward went on as usual. Nurse Hilary, with her cheerful voice and pleasant manner, went about her daily work, spending most of her time beside the screened-off bed where No. 10 (white, weak, and very silent now) lay, coming back to life and strength, feebly smiling her thanks up to the face as white as her own that bent above her. A tired, pale nurse's face is no uncommon sight; yet Lady Beckett, treading on weighty tiptoe into the ward one morning, her roseate face beaming under the dandelions, and over an armful of peonies and periodicals, was shocked with the wan and haggard one that greeted her. She trotted downstairs, interviewed the kindly Matron, and that very afternoon Hilary found herself in a long hammock chair in her ladyship's garden by the river; flowers about her, sunshine above her, and the knowledge that rest—rest such as only life's toilers can know—was to be hers for ten days.

When she went back to her work again No. 10 had gone, and a new suffering face lay on No. 10's pillow.

Two years had passed. It was a clear warm day at the end of May. The little garden at the back of St. Elizabeth's was bright with sunshine dancing through budding leaves, and sweet with the scent of lilac blooms.

There had been many such days that May, and the patients lying or sitting amongst these spring delights had almost grown accustomed to the transformation from ward life all day to premature summer out of doors, and yet this was to be an eventful day nevertheless.

Under the heavy lilac branches Hilary, Sister Hilary now, stood by a long chair in which lay a worn-faced, fair-haired woman. Weak, ill, almost starving, she had come to the hospital only that morning, and Sister Hilary had quickly recognized her as Mrs. Webster, the No. 10 of two years ago.

"I heard from him last week, he has been in the Soudan; he may be home any day now," she was saying; "he has had a hard time of it, but he will make a fresh start now, and I shall soon get well when he comes."

"Yes, we must have you quite strong again to meet him," answered Hilary, gently.

Many feelings were at war within her as she spoke—love, sorrow, conquered bitterness, and, above all, pity, deep and real, for the helpless little woman. Her strong protective nature was stirred to its very depths, and her head was very erect, and her eyes shining with a warm light, as she turned away and left the garden.

As she crossed the cool entrance hall a man suddenly stood in the open doorway, a glow of scarlet against the strong sunlight pouring through it.

Sister Hilary stood quite still; and in a breathing space Alan was before her. How well she knew that sturdy figure, that sunburnt face, with its frank smile; and yet she did not move or speak.

He took her hand in silence, and the eager gladness on his face faded.

There was a look almost stern in its gravity on her face; then she said, "Will you come this way? She is expecting you," and went rapidly out at the garden door before him. The patients stared, as well they might, as the two passed. Sister Hilary stepping quickly over the grass, quiet, dignified. Her soft grey dress, little blue cape, and white cap were not unlike a bit of the spring sky above her—and Alan in scarlet and gold shining gloriously in the sun, with the little plain cross inscribed with "For Valour" glinting on his breast.

"It'll likely be her sweetheart, come back fro' them Sudden wars," observed an interested old woman to her neighbour, "them with the He-gipsy-uns," she added, by way of explanation.

"Your husband is here," said Sister Hilary in a low voice.

Alan's face wore a startled look, and Mrs. Webster turned quickly.

What did this mean? No cry of joy, no sob, no clinging hands? A smile of hearty pleasure only, between the two; a handshake as of old friends, a—

"Why, Nelly, how did you come here?" from Alan, and

"But where is my husband?" from the pale little woman in the chair.

"He'll be here soon," said Alan, "meanwhile"—

He turned to Hilary. Her face was white, her eyes shining, her hands outstretched.

"Come away, dear," he said, "there has been some terrible mistake."

(*To be continued.*)

### Appointment.

[It is requested that successful candidates will send a copy of their applications and testimonials, with date of election, to The Editor, The Lodge, Porchester Square, W.]

Royal South Hants Infirmary.—Miss Hedwig Ida Proschivitzky has been appointed head nurse of the male accident ward of this infirmary. Miss Proschivitzky trained at the Marylebone Infirmary, and for the last three years has been head nurse at the Darlington Hospital.

### Amusements and Relaxation.

### SPECIAL NOTICE TO CORRESPONDENTS.

First quarterly word competition commenced January 3rd, 1891; ends March 28th, 1891.

Competitors can enter for *all* quarterly competitions, but no competitor can take more than *one first prize* or *two* prizes of any kind during the year.

Proper names, abbreviations, foreign words, words of less than four letters, and repetitions are barred; plurals, and past and present participles of verbs, are allowed. Nuttall's *Standard* dictionary only to be used.

N.B.—Word dissections must be sent in WEEKLY not later than the first post on Thursday to the *Prize Editor*, 140, Strand, W.C., arranged alphabetically, with correct total affixed.

The word for dissection for this. the NINTH week of the quarter being "PRIMULAS."

| Names. | Feb. 19th. | Totals. | Names. | Feb. 19th. | Totals. |
|---|---|---|---|---|---|
| Reynard | — | 77 | Woodbine | — | 25 |
| Reldas | 94 | 393 | Madame B. | — | 25 |
| Tinie | — | 30 | Shakespeare | — | 59 |
| Patience | — | 76 | Smyrna | 73 | 259 |
| Jenny Wren | 87 | 346 | Southwood | — | 102 |
| Agamemnon | 92 | 383 | Gipsy Queen | — | 21 |
| Wyamaris | 85 | 369 | Snowball | — | 19 |
| E. C. | 89 | 384 | Rita | — | 88 |
| Ecila | — | | Mortal | — | 16 |
| Hope | 9[illegible] | 387 | Nurse Annie | — | 15 |
| M. W. | 89 | 380 | Carmen | — | 11 |
| Qu'appelle | 87 | 376 | Grannie | — | 45 |
| Nil Desperandum | 93 | 386 | Amie | — | 30 |
| Lady Betty | 90 | 364 | M. R. | — | 25 |
| N. A. S. | 85 | 324 | Primrose | — | 24 |
| Sister Jack | — | 62 | Nurse J. S. | 81 | 181 |
| Crystal | — | 203 | B. A. C. | 59 | 102 |

### Notice to Correspondents.

N.B.—Each paper must be signed by the author with his or her real name and address. A *nom de plume* may be added if the writer does not desire to be referred to by us by his real name. In the case of all prize-winners, however, the real name and address will be published.

